# Automated noise modelling using a triangulated terrain model

**DOI:** 10.1080/10095020.2023.2270520

**Published:** 2023-12-05

**Authors:** Nadine Hobeika, Laurens van Rijssel, Maarit Prusti, Constantijn Dinklo, Denis Giannelli, Balázs Dukai, Arnaud Kok, Rob van Loon, René Nota, Jantien Stoter

**Affiliations:** a 3D Geoinformation, Delft University of Technology, Faculty of Architecture and the Built Environment, Delft, The Netherlands; b 3DGI, Zoetermeer, The Netherlands; c National Institute for Public Health and the Environment (RIVM), BA Bilthoven, The Netherlands; d Ministerie van Infrastructuur en Waterstaat (IenW), Den Haag, The Netherlands

**Keywords:** Noise modelling, geomatics, TIN, CNOSSOS EU Directive on Noise Assessment, 3D urban applications

## Abstract

Noise simulations are an important part of noise studies that investigate the impact of noise sources on the environment. In noise simulation, noise levels at receiver points are calculated based on the noise propagation paths between the receiver and source points. These paths are derived from the height of the terrain. In current calculation approaches implemented in noise simulation software, 3D polylines are used as input to describe the height of the terrain. These 3D polylines are semi-automatically generated to meet the highly demanding computing performance of simulation software. In addition, previous research showed that the reconstruction of appropriate height lines as used in noise simulation is very difficult to automate, if not impossible As a solution, this research investigates how noise propagation paths between receiver and source points can directly be generated from a Triangulate Irregular Network (TIN) without creating the height lines. This would allow us to use the automatically generated TIN as input for noise simulation instead of the height lines. In addition, a TIN enables better control of the quality of the data than height lines do. This study uses the 3D noise modeling guidelines of Common Noise Assessment Methods in Europe (CNOSSOS-EU). Algorithms have been developed and implemented in a prototype to generate and validate the paths between receiver and source points using a TIN that includes the buildings as well as the noise absorption properties of the terrain. The prototype is successfully tested on two scenarios from the Netherlands. Since CNOSSOS-EU guidelines were used, the prototype is applicable to the entire European Union and can be the first step in improving the automation of 3D noise modeling using currently available techniques and data.

## 1. Introduction

Nowadays, cities are facing several challenges to provide their citizens with a healthy urban environment. A particular threat to a healthy city is noise pollution. Currently, 55% – and increasing to 68% by 2050 – of the world population lives in urban areas where noise pollution has a negative effect on people’s health and the environment (United Nations 2018).

Europe is taking fundamental measures to fight noise pollution. The Environmental Noise Directive (2002/49/EC) requires EU Member States to determine the exposure to environmental noise through strategic noise mapping and elaborate action plans to reduce noise pollution (European Commission n.d.). Annex II of this directive specifies the assessment methods that should be used by Member States for noise measurements and computations, i.e. the Common NOise aSSessment methOdS (CNOSSOS-EU) for road, railway, aircraft and industrial noise (DIRECTIVE 2002/49/EC 2002). These assessments include methods to simulate noise propagation between source and receiver points in order to assess how the proposed action plans will reduce the noise pollution in a certain area.

The prescribed methods to calculate noise levels are implemented in the current, mostly commercial noise simulation software. 3D polylines are used as input that describe the terrain profile with as few height lines as possible to meet computation performance demands. This has several drawbacks. Firstly, height lines are not capable to capture all relevant height details between the receiver and the source points, since it does not reflect height differences that occur between two lines. Representing all relevant height details of the terrain that contribute to the propagation of noise levels will make noise simulation more reliable, specifically near the noise source. A second drawback of the currently used 3D height lines is that they are difficult to be generated in an automated manner from available height data. To reduce the size of the input data as much as possible, height lines are generated at positions where height variance is high. In addition, more lines are needed near the noise source, considering that the closer the height line is to the source, the greater its relevance to the model. The required human interaction to generate such lines hinders both the automation and standardization of 3D noise modeling. Thirdly, it is hard to guarantee or control the quality of the semi-automatically generated height lines.

### 1.1. 3D height lines versus TIN: motivation of this research

From the research conducted by (3D geo-information 2020; Stoter et al. 2020) aiming to generate these height lines as input data for noise simulation automatically, it was concluded that extracting the required height lines from a TIN is not a satisfactory technique to substitute the semi-automatic approach of height lines reconstruction. Moreover, the original TIN (the most common and accurate way to capture terrain heights, see Section 2.2.3) – from which the height lines are derived in the automated process preserves the height data most accurately. Using the TIN directly in noise simulation would therefore eliminate the need for all the conversion steps to satisfy the input requirements of current simulation software. In addition, a TIN also offers a suitable data structure for computations and for finding the optimal balance between simplification (beneficial for computing performance) and height line accuracy. Finally, a TIN can be stored efficiently by storing the points of the terrain in a set order, e.g. the points of the triangles, followed by pointers to adjacent triangles and finally to semantic information. This makes the TIN stand out compared to other data structures.

These findings were the motivation for the research presented in this paper, which investigates the feasibility to use the TIN as input for 3D noise modeling using the Netherlands as a case study. A prototype was developed as a proof of concept for *using a triangulated terrain model in noise simulation to allow for automated 3D noise modeling in accordance with the guidelines of CNOSSOS-EU*. In the prototype, the possibilities to automatically generate robust input data describing the height of the terrain in a TIN are aligned to algorithms for noise simulation that generate noise propagation paths from noise sources and determine noise levels at receiver points. As results will show, the prototype is able to generate noise propagation paths between receiver and source points directly from the TIN by which a TIN can indeed serve as input for noise simulation. In future work, the algorithms can be further developed to be implemented in noise simulation software. This would enable input data describing the height of the terrain to be fully automatically generated and lead to more consistent and reliable simulation results as well as cost reduction in noise studies.

### 1.2. Overview of this paper

This paper describes the research that developed, implemented and evaluated a prototype for a TIN-based noise simulation. The paper will first present an overview of relevant work and will also detail the main concepts of this work: noise modeling, TIN and CNOSSOS EU in Section 2. Section 3 will present the methodology to prove the concept of TIN-based noise simulation. The development and implementation of the prototype is described including the decisions made during this process as well as the evaluation method of the TIN-based noise simulation prototype. The main part of the evaluation consists of comparing noise maps obtained from the prototype with noise maps obtained from an existing line-based implementation for two scenarios. The results of the evaluation as well as recommendations for future developments are discussed in Section 4. The paper will end with conclusions in Section 5.

## 2. Related work and background

### 2.1. Related work

Several approaches for noise modeling have been developed and tested before. Nowadays 95% of the noise maps are visualized in 2D and 5% in 3D (Pervez et al. 2020; Stoter, Kluijver, and Kurakula 2008) presented the limitations of 2D approaches for noise modeling especially for urban areas and argue that 3D approaches yield better accuracy for different floors of a building. According to (Bocher et al. 2019), noise modeling is still more of a 2D approach that still needs to account for ground type and road section types. The 3D approach presented by Stoter, Kluijver, and Kurakula (2008) mainly consists of interpolation of noise levels at different height levels of the 3D city model and then overlaying these interpolated (2D) surfaces on top of the 3D model. This work implements the Dutch calculation methods for noise the “Standard Calculation Method 1” (RIVM 2014). Beran et al. (2021) propose and study several new methods for visualizing the third dimension of continuous phenomenon, such as noise. All visualizations in this research are based on the use case of road-traffic-generated noise in outdoor urban areas. Another initiative to model noise from traffic in 3D has been done by Zhao et al. (2017). In this work, the buildings are modeled via a triangulated surface mesh allowing for complex structures, but featuring only direct, line of sight, propagation paths.

In Farcas and Sivertun (2012), tools in the ArcGIS environment were created to produce noise maps in 2D as well as in 3D for a larger region. In 2019, Bocher et al. (2019) implemented a 2D noise modeling plugin for ArcGIS to improve data visualization of noise modeling. However, it does not use a standardized method and it aims at producing noise maps from a limited amount of information, leading to simplified paths and terrains which could result in large errors in noise levels, especially in areas of varying heights. Such approaches are specifically suitable for global assessment studies where limited data are available.

Standards, methods and (quality of) input data for noise simulation differ across EU member states (Licitra 2012). Therefore, as was already described in the introduction, the European Union 2002/49/EC Environmental Noise Directive (END) requested a common noise assessment method and standardized guidelines. In 2015, the request was granted with the European Directive 2015/996 which introduced the CNOSSOS method to be used for the periodical noise mapping from 2022 onwards. This method describes how to determine the noise level for a given propagation path. Apart from the software implementations for CNOSSOS (see Section 2.2.4), little has been done for the practical guidelines outlining the specifications for the required input data, metadata, and the schema design to test the real-world situations with CNOSSOS. Therefore, Kumar et al. (2020) propose a harmonized data model for modeling input and output data for CNOSSOS-compliant noise simulations, and they also generate a real-world data set based on their data model for simulating urban noise using CNOSSOS.

Marking the importance of having noise modeling guidelines, the END was added to the international 3D standard CityGML through the CityGML noise ADE (Application Domain Extension). Kumar et al. (2017) later point out the limitations of the CityGML extension such as missing the noise emitted from industry, missing information about noise barriers, and missing information about the vehicles’ speed. As a solution, Kumar et al. (2017) supplemented the ADE with new classes and attributes. Another guideline implemented is the French national method “NMPB-08” in the open-source GIS plugin NoiseModelling.

Different methods have also been suggested to capture noise levels from the field. Picaut et al. (2018) have suggested a crowdsourcing approach to measure noise levels in the field that uses a smartphone application to measure noise and aggregate data collected to output a noise map. Their open-source application NoiseCapture does not only collect noise levels but also the pleasantness of the noise as perceived by the volunteering users. Field measurements are relevant, although they are not reliable to measure the noise level produced by a specific noise source due to the presence of background noise. In addition, noise simulation is required to determine and assess the noise levels of future situations such as different alternatives of a highway or the construction of noise barriers. Therefore, in noise studies simulations are prevailed above field measurements.

The work of Stoter et al. (2020) shows how to automatically generate input data (i.e. buildings, height of the terrain, noise absorption/reflection areas) for a standardized noise simulation method from available open data. The used data sets are the national height model of the Netherlands (AHN) represented by a point cloud data set, the building and address registry of the Netherlands (BAG) represented by a polygon data set, and the registry of large-scale topographic objects represented by the geometry and semantics of these objects. The last data set is used to identify whether a terrain area is noise reflective or noise absorptive. As explained in the introduction, the study suggests using a TIN as a future development for their project, which was the motivation for this research.

### 2.2. Main concepts of this work and motivation to explore TIN-based noise simulation

#### 2.2.1. Noise modelling, noise simulation and noise assessment

Noise propagation modeling is the calculation of noise levels produced by a noise source at receiver points by applying noise propagation algorithms. “Noise simulation” is often used in a more narrow context as it refers to the process of using software that implements the noise modeling method to calculate noise levels at receiver points. In this paper, the terms “noise modeling” and “noise simulation” are used interchangeably. Interpolating the estimated noise levels at discrete points – which are the output of noise simulation software – to continuous noise maps and assessing the impact of these interpolated noise levels on the environment, like the number of people that are hindered by noise levels exceeding certain threshold values, is what is called noise assessment.

**Figure 1.**
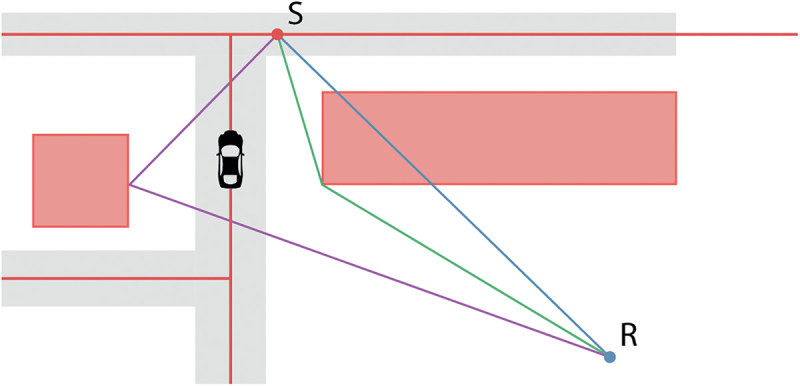
Three types of propagation paths from source to receiver; reflected in purple, diffracted in green and direct path in blue. The roads are coloured red as is the source point.

Noise modeling uses input data containing relevant information about the environment and consists of two consecutive steps: first finding propagation paths between source and receiver points (located at positions of interest) and secondly, deducing the noise level from these paths at the receiver caused by that source and the noise effects (absorption/reflection) along the propagation path. The propagation path consists of a vertical cross-section of the terrain plus the buildings that are on top of the terrain along horizontal propagation paths that takes into account the building facade details. These paths can be direct, reflected or diffracted paths along buildings, as shown in Figure 1.

#### 2.2.2. Digital terrain modelling (DTM) and digital surface modelling (DSM)

A DTM is a digital representation of only the bare ground elevations excluding any other objects such as buildings or trees. A DSM represents ground elevations including all other objects that can be found on a terrain (Ledoux et al. 2020). They both provide information on the elevation of a terrain and can be described by a TIN-based data structure.

#### 2.2.3. TIN

A triangulated irregular network (TIN) is a network of interconnected (usually) Delaunay triangles, which combined, creates a two- or three-dimensional 2-manifold, i.e. it can be “unfolded” to a flat surface without changing the topology (connections) (Ledoux et al. 2020). Using triangles to represent a height surface has several benefits for the noise propagation modeling, namely;

1. It is the simplest shape to describe a 2D plane in 3D space.
2. The triangulation of height points – the format in which terrain height is commonly surveyed, by, for example, Light Detection And Ranging (LiDAR) technology – is an accurate and size-efficient method to model the height of the terrain. The creation of a TIN draws triangles with variable size and shape over the terrain. The triangles follow the terrain surface – as captured in points – as most accurate as possible.
3. A TIN can be optimized (i.e. simplified) to use few triangles to represent flat areas and use more triangles to accurately represent complex structures like ditches, hills or rock. Using this principle, a terrain can be modeled with a certain guaranteed accuracy, defined as the maximum error the model is allowed to have compared to the ground truth. When the maximum acceptable error is high, it will represent the terrain with fewer triangles, but still represent complex formations relatively accurate. When the error threshold is low, it will more accurately follow the terrain, with more triangles. This allows simplification to meet computation performance challenges while keeping the desired accuracy. In addition, TINs of varying accuracy of the same area are exactly the same in use, and can therefore be used interchangeably, allowing for quick processing of both larger areas (at lower accuracy) and smaller, detailed areas for local studies (at higher accuracy).
4. The terrain can be represented as points in a two-dimensional surface, each holding a height value and additional required information to be processed later, in this case building heights and material type. Therefore, a cross-check between the TIN, a buildings data set, and a data set containing ground types is no longer needed as is the case when the three layers are used as separate input layers for noise simulation (which is current practice). Eliminating the cross-check step allows for both more consistent and time-efficient noise simulation algorithms.
5. All edges of the triangles are incident to the edge of the triangle next to it, unless it lays on the edge of the model. This relation can be stored in a triangular data structure where each triangle has an ID, three vertices and, respectively, three neighbors. This structure can be used to “walk” over the triangles, while following a line or a direction, as required to derive noise propagation paths.

In conclusion, the advantages of a TIN for noise simulation are as follows: it enables to control the quality, it is a robust, size- and computation-efficient data structure and it enables to easily derive a vertical cross section, by “walking” over the triangles to obtain noise propagation path as required in noise simulation. Assuming the TIN is extracted from representative information like a point cloud, it can be assured that the cross-section follows the terrain with a maximum allowed error of the defined threshold at which the TIN is generated.

#### 2.2.4. Test_Cnossos

Test_Cnossos (Cnossos-EU-SWE 2020) is an open-source implementation of the CNOSSOS-EU guidelines for noise calculations that were used to assess the prototype. The software calculates noise levels at receiver points for prepared propagation paths and energetically adds up the noise levels for the propagation paths at each receiver. The guidelines allow for variation of the determined propagation paths.

Test_Cnossos requires as input an XML file of the propagation path from the source point, passing through a reflection point if applicable, to the receiver point. The propagation path is a set of consecutive 3D-coordinate points along with their respective material. This path represents the cross-section of the DSM between the source and receiver points. The material stored with a point is a letter that refers to an absorption index defined by CNOSSOS-EU for different types of material. Meteorological conditions influencing sound propagation are also written to the path based on preset values. The output of Test_Cnossos is an XML file with the noise level at each receiver of the propagation paths

## 3. Methodology

To provide a proof of concept for the hypothesis that noise modeling can be performed directly on a TIN, a prototype has been developed, see Section 3.1. Two methods to evaluate the prototype are described in Section 3.2.

### 3.1. Prototype for TIN-based noise simulation

The developed prototype finds noise propagation paths from the TIN from noise source points – that represent a linear noise source like a road – to a set of receiver points and transfers these into cross sections. The TIN includes buildings as well as noise absorption/reflection characteristics of the terrain. In a next step, Test_Cnossos is used to calculate the noise levels at each receiver point from those generated cross sections. Three types of noise propagation paths have been implemented: direct paths, vertical diffraction and first-order reflection paths since these three together determine the major part of noise levels at a specific point.

The development of the prototype consists of five steps which are described in the following subsections:

1. A TIN containing the required information was generated that contains the required information, see Section 3.1.1
2. Receiver points were generated, see Section 3.1.2
3. For each receiver point, the noise source determining the noise level at that point was found, see Section 3.1.3
4. The core of the prototype is an algorithm that generates the cross-sections (i.e. propagation paths) between the source point and the receiver point from the TIN. This is described in Section 3.1.4.
5. Finally, the noise level at each receiver point is determined with Test_Cnossos using the cross sections outputted by the developed noise propagation algorithm as input, see Section 3.1.5

#### 3.1.1. Generation of the TIN

**Figure 2.**
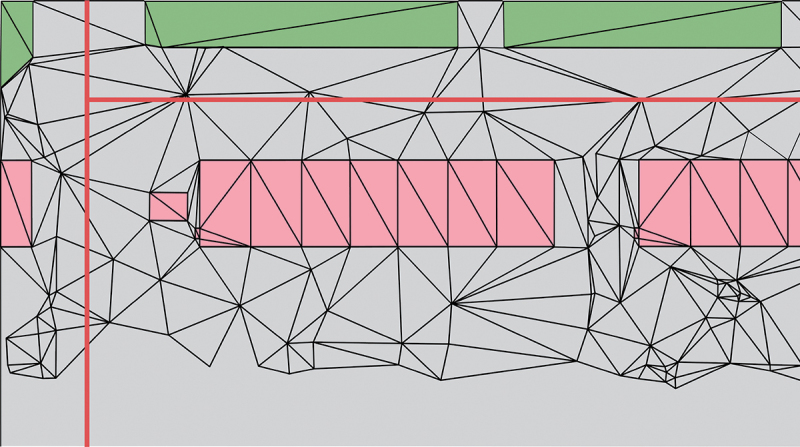
LoD2 TIN constrained to buildings (pink) and ground types (green), with roads as red lines.

In this step, a LoD2 TIN was generated as a Digital Terrain Model with semantic information about objects integrated in the terrain, according to the LoD specifications for terrains proposed in Kumar et al. (2019). In this case, the integrated semantics are those required for noise simulation. The LoD2 TIN is thus constrained to building footprints and ground-absorption/reflection areas converted from land use areas (Figure 2) and the triangles are semantically enriched with the height of the building or their ground type (absorbing or reflecting) in case the ground is not covered by a building.

The heights of the buildings are obtained from so-called LoD1.3 buildings (Biljecki, Ledoux, and Stoter 2016). In this representation, buildings are extruded to either one height or multiple heights in case of significant height differences like a church with a tower or a building with a shed attached.

**Figure 3.**
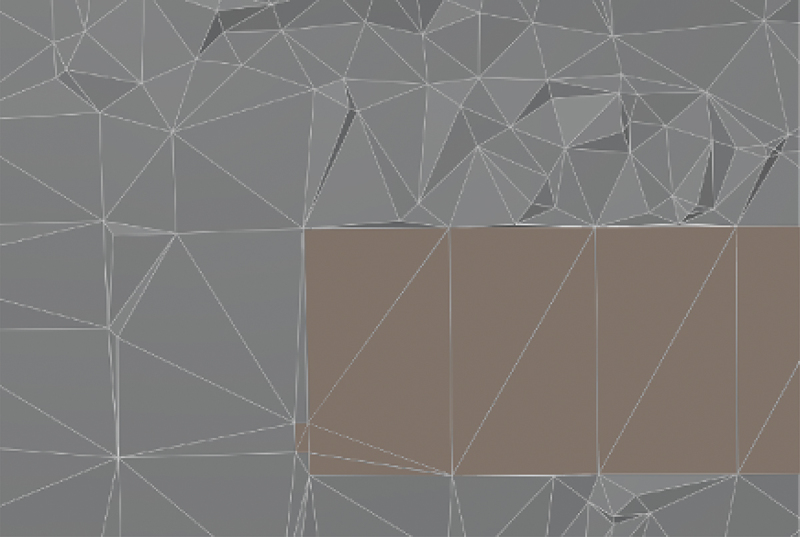
LoD2 TIN with buildings embedded.

The prototype algorithm supports thus three material types that are semantically embedded in the TIN: building height, noise absorbing ground, noise reflective ground. The height of the TIN remains at terrain level at the location of the building. The height of the building is an attribute of the triangle and processed during the cross-section generation, see Figure 3.

#### 3.1.2. Generation of receiver points

Multiple receiver points must be defined to get a representative noise map at the end. A set of equidistant receiver points was created to output a homogeneous noise map for assessment and analysis in a later stage. Receiver points inside buildings were removed.

#### 3.1.3. Detection of noise source for each receiver point

**Figure 4.**
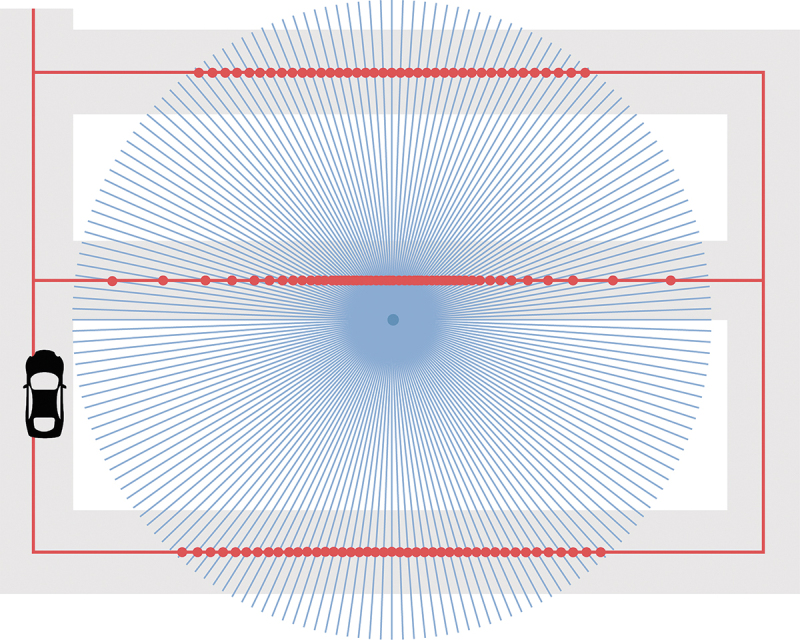
Finding the source points by drawing two-kilometer rays from a receiver point at a two-degree interval and extracting their intersection with roads (red: the roads and the source points, blue: the rays for one receiver point).

In the prototype, roads represented by their center lines are used as noise sources. According to CNOSSOS-EU, only sources within a 2 km radius from a receiver need to be taken into consideration. To detect the noise source for each receiver point, two-kilometer-long rays are shot from each receiver point at a two-degree interval and the intersection with the road lines creates all source points that contribute to the noise level for that specific receiver point, see Figure 4.

**Figure 5.**
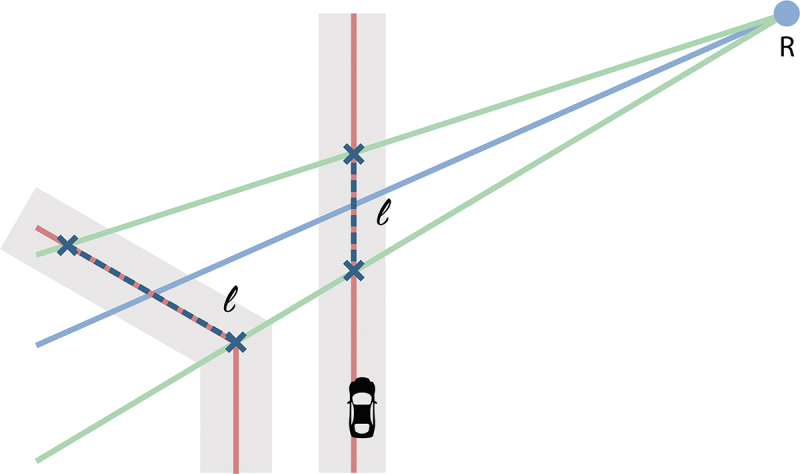
Estimation of the road length to determine the weight of a source noise (red: road lines, blue dot: receiver, green: one-degree rays on both sides of the intersection line used to estimate the respective road length, blue dotted lines: estimated road length).

Source points are then weighted depending on the portion of the road length they represent, as can be seen in Figure 5.

The CNOSSOS-EU guideline converts line road sources into point road sources based on an equidistant division of the road instead of finding them via the location of receiver points. But the principle is the same and the approach of this research was easier to implement.

#### 3.1.4. Derivation of propagation paths

The core of the prototype is an algorithm that detects the propagation paths between source and receiver from the TIN. These paths consist of a list of 3D coordinates with their material type attached to these (’building height’, noise absorptive or noise reflective).

The CNOSSOS-EU guidelines (European Commission n.d.) indicate four types of elementary paths to be considered for noise modeling, namely:

- Type 1 – “Direct” paths, including vertical diffraction
- Type 2 – Paths reflected on vertical (or slightly sloping) obstacles
- Type 3 – Paths diffracted by lateral edges of obstacles
- Type 4 – Mixed Paths.

Among these four types of propagation paths, the algorithm developed in this research takes into account the first two types since these cover the most noise-relevant cases:

1. Type 1 paths are “‘direct’ paths from the source to the receiver, which are straight paths in plane view and which may nevertheless include diffraction on the horizontal edges of obstacles” (see Figure 6) (European Commission n.d., 81/180)

**Figure 6.**
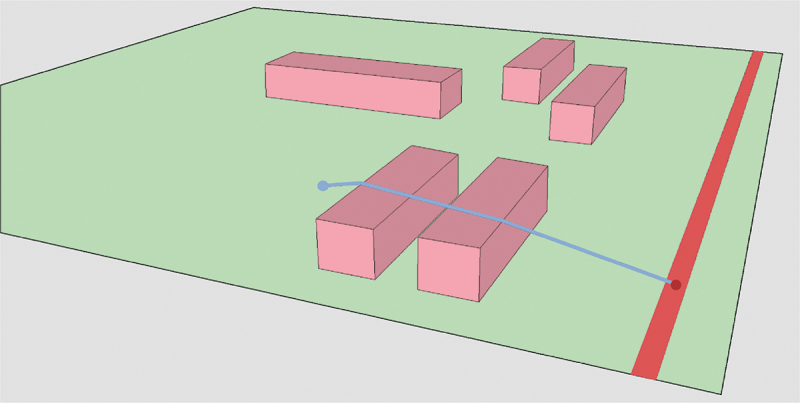
Example of type 1 path (direct path). Adapted from CNOSSOS-EU.
2. Type 2 paths, are the paths “reflected on vertical or slightly sloping (<15°) obstacles (…)” and “may also include diffraction on the horizontal edges of obstacles (…)” (see Figures 7 and 8) (European Commission n.d., 81/180)

**Figure 7.**
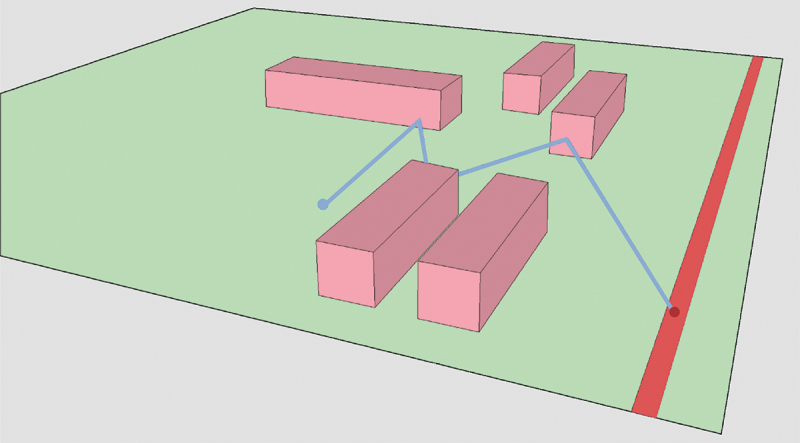
Example of type 2 path (reflection path) without diffraction. Adapted from CNOSSOS-EU.

**Figure 8.**
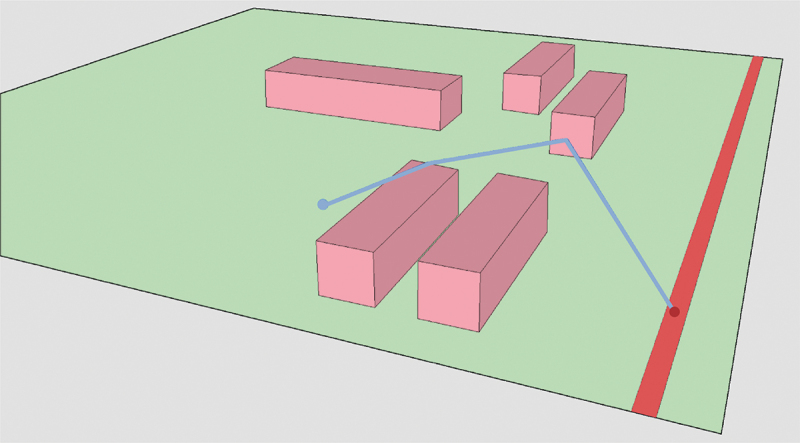
Example of type 2 path (reflection path) with diffraction on horizontal edge. Adapted from CNOSSOS-EU.

##### 3.1.4.1. Identification of the receiver triangle

The derivation of the propagation path starts by finding the triangle in which the receiver lies. To find this triangle, the “walk” algorithm is used (Zemek 2014). The algorithm starts at a triangle (T) in the TIN. Using orientation calculations on the edges of T and the position of the receiver point, the algorithm determines which incident triangle to T the walk algorithm needs to go to next to get closer to the receiver point. This process is repeated until the walk algorithm reaches the triangle that the receiver point is located in.

It is computationally expensive to find the triangle in which a point is located. Therefore, the operation should be performed as few times as possible. Since there are fewer receiver points than source points, it is more efficient to find the starting triangle (with the source point) for each receiver point. This is what was implemented.

##### 3.1.4.2. Generation of reflection points

**Figure 9.**
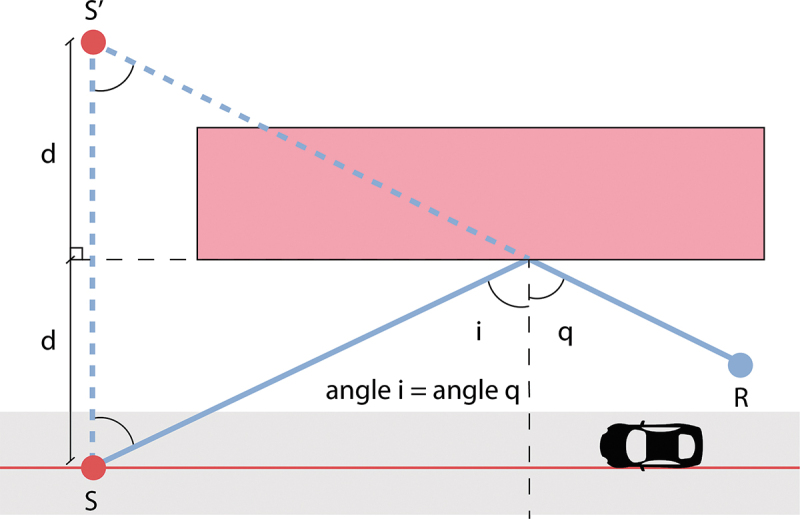
Example of determining the reflection point on an obstacle through the image source method (S: source, S’: image source, R: receiver). Adapted from CNOSSOS-EU.

The reflection points are found with the “image method”. This method uses every wall in a building within a 2 km radius as a mirror to create a virtual image (S’) of the source point (S). If the S’-receiver segment intersects the wall that acted as a mirror, a candidate reflection point is created, see Figure 9.

**Figure 10.**
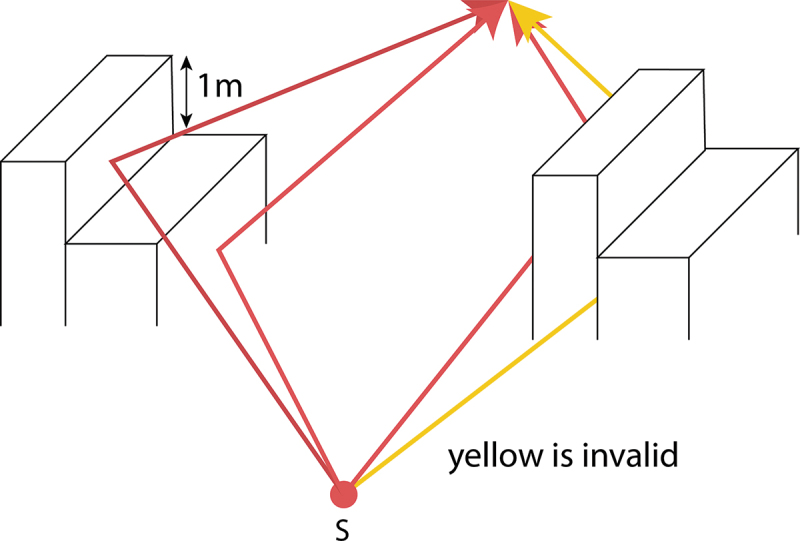
Second validity test reflected paths; verifies that if the reflecting wall is a common wall of two buildings, the building on the exterior of the reflecting building is at least 1 meter lower than the reflecting building. The height of the source or receiver has no influence on the test. The red paths are valid, the yellow path is not valid and therefore not further processed by ignoring its associated generated reflection point.

Before the candidate reflection point is added to the simulation that point undergoes two extra validity tests:

- Check if the building that generated that point is large enough to reflect noise
- Check if the reflection point lies on two incident walls that have a substantial height difference (larger than 1 m) to only include valid reflections and to ignore the detected candidate reflection points that have no impact, see Figure 10.

The candidate point is then added by creating a list of paths that are tested and valid, and then the algorithm goes over each path to produce the cross section.

##### 3.1.4.3. Straight walk

**Figure 11.**
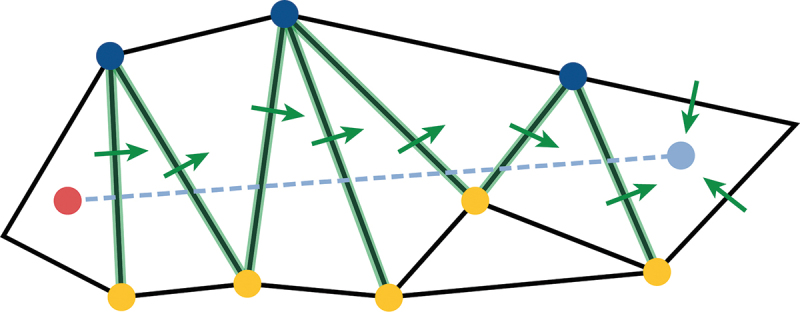
Straight walking to the receiver triangle.

To create a cross-section, a straight path is required from the receiver point to the source point. The straight walk algorithm (Zemek 2014) is used again to derive this path, see Algorithm 1 in the Appendix and Figure 11. To start the algorithm, the receiver point, source point, and starting triangle, which is the triangle the receiver point is located in, are needed. At each iteration of the walk, the edge of a triangle that the straight path intersects with next is returned. This is done by conducting a side-test between each vertex of the edge and the receiver-source segment. If an edge is collinear with the origin-destination segment, the intersection point is set half-way between the two end-points of the edge. The process is repeated until the walk has arrived at the destination point. This provides a list of all edges that the straight path intersects on its walk from receiver point to source point. These edges are used to make the cross-section.

##### 3.1.4.4. Generation of noise propagation paths

There are two types of cross-sections to construct:

- Direct path cross-sections, from receiver to source;
- Reflected path cross-section, from receiver to reflected point to source.

The reflection point and source point are stored in a list of destinations that need to be looped over. The origin is set to the receiver at first and then, when looping through the second destination, the origin is set to the reflection point. Creating the cross-section between source and receiver point as a list of 3D coordinates, with the material type attached to them, is done during the straight-walk algorithm of (Zemek 2014) that was explained above. Thus, the walk-algorithm is used two times: to get the start triangle and to get the semantically enriched cross section.

The generated TIN (see Section 3.1.1) is used as input for the straight-walk algorithm. The TIN contains information about neighboring triangles which makes it possible to derive a cross-section by walking over the triangles. As described above, the TIN also contains information on the height of the buildings and absorption/reflection characteristics so that this information can be assigned to the intersection points forming the cross-section.

For every intersection point, its height is interpolated using the side test that was used to find the designated edge to assign weights to the end-points of the edge. Before adding that point to the cross-section, the point first needs to be provided with semantics (see next step: “Addition of Semantics”). The interpolated, semantically enriched point is added to the cross-section and then the algorithm keeps walking. When the algorithm reaches the destination triangle, the ground height of the source is interpolated in the TIN and it is appended with the material of the triangle. In the end, the order of the vertices in the cross-section is reversed to have a source-receiver cross-section. A simplification is applied by not adding a point in the cross-section if that point lies within 10 cm (3D Manhattan distance, sum of the distance per axis) of the previous point.

##### 3.1.4.5. Addition of semantics

Since the straight-walk algorithm moves from receiver to source, and not from source to receiver, the intersection point inherits from the next triangle on the straight path instead of the current triangle to adhere to the CNOSSOS-EU guideline.

If the current triangle and the next triangle are both ground, each intersection point inherits the ground material of the next triangle. For this, the absorption index of the triangle is checked and the intersection point is assigned a letter, “C” or “G”. “C” stands for absorption since – according to the CNOSSOS-EU guideline – it refers to “uncompacted, loose ground (turf, grass, loose soil)” with an absorption index of 1.0 and a sigma of 80. ’G’ stands for reflection according to the CNOSSOS-EU guideline and refers to ’hard surfaces (most normal asphalt, concrete) with an absorption index of 0.0 and a sigma of 20,000 (van Maercke 2012). Furthermore, since the data sets provided have accuracy limitations, it was decided that any difference of plus or minus 10 cm between two points of the same material is superfluous in the cross-section. As a consequence, a threshold was set to skip these close points.

**Figure 12.**
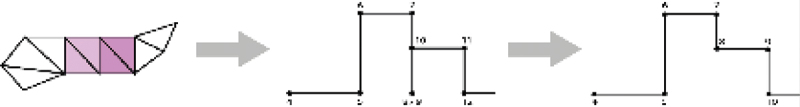
Embedding the height of buildings in the cross-section (purple/pink triangles indicate a building) and adding the semantics if a point is on ground and it corrects for overlapping wall parts.

When a building is reached, the cross-section needs to go up, which means both the intersection point and the lifted intersection point to roof level, with their corresponding building material, are added to the cross-section. In the code, all building materials are given an ’A0’ letter according to the CNOSSOS-EU guideline. A marker is also added to indicate that a cross-section is in a building. This marker helps to avoid adding intermediate points that are not part of the DSM cross-section before going down again (Figure 12). If the cross-section is already in a building and the next triangle is the same building or has the same height (plus or minus 10 cm) and the same material, then the cross-section ignores the corresponding intersection points.

If the cross-section is already in a building and the next triangle has a different roof height, two points are added, each at the height of the current and the next triangle. If the cross-section is already in a building and the next building is ground, both the lifted point with the building material and the intersection point with the ground type are added to the cross-section. To make the code more robust, a condition to skip a building that has a roof level lower than the ground TIN level is added.

##### 3.1.4.6. Optimisation with collinear sources

**Figure 13.**
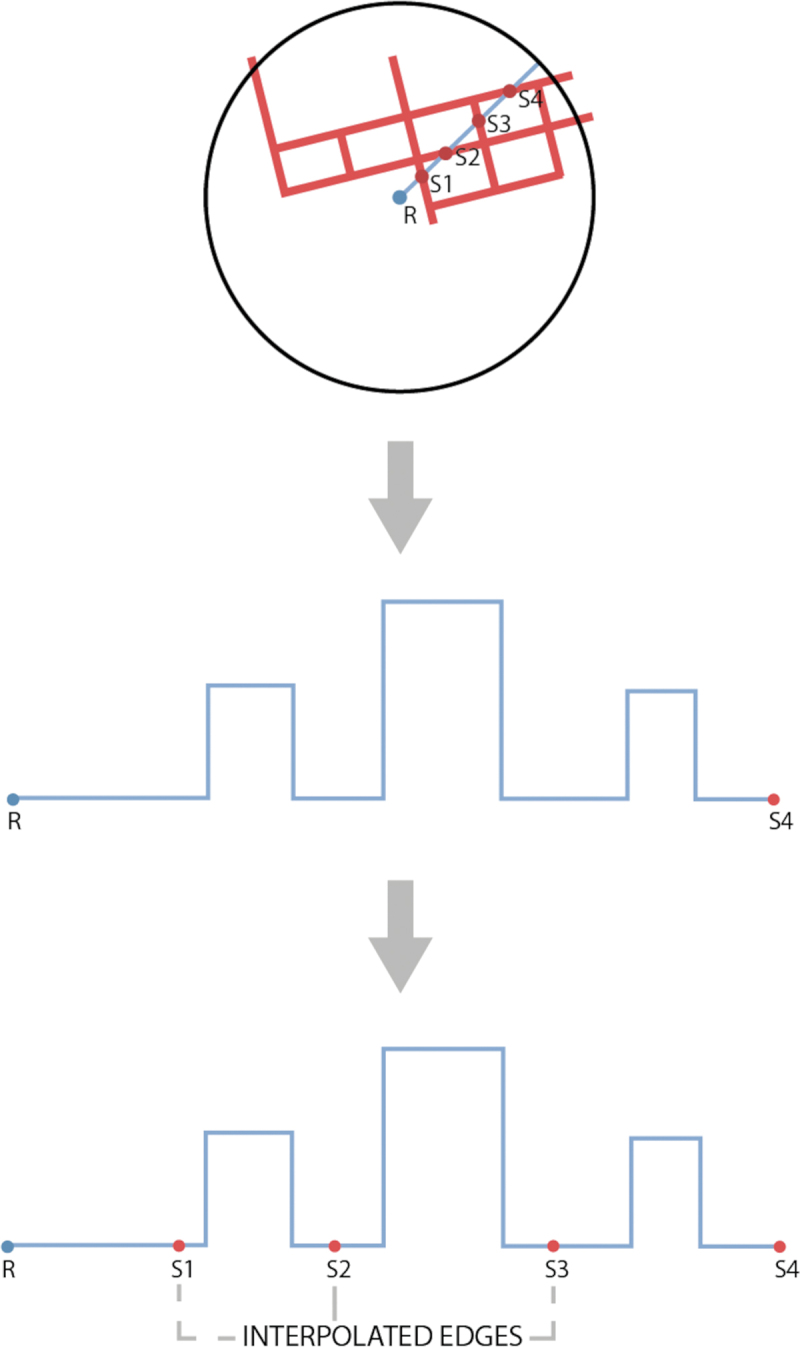
Linear interpolation at the edges, red lines are road, s1 to s4 are collinear sources.

Since sources are created by intersecting rays from a receiver, constructing cross-sections can be optimized by walking from receiver to the furthest source point along a ray and then each source point along the path splits the cross-section. To split the overall cross-section, 2D sources are located in the overall cross-section, and their heights are linearly interpolated from the edges in that cross-section (Figure 13). Weights are assigned to each endpoint of an edge according to the distance along an axis between those points, respectively, and the interpolated point.

##### 3.1.4.7. Export of the cross-sections

The derived cross-section is stored as a list of Cartesian points (in Euclidean space), supported by their materials and extensions. In order for the Test_Cnossos software to process this cross-section, it should be written to an XML file, according to requirements from the JRC-2012 calculation method mentioned in van Maercke (2012). However, to produce a valid XML file, a few changes are required. These changes, which are described in Algorithm 2 in the Appendix, are needed to produce a valid and correct XML file. The source, receiver and optionally wall extension are added to the respective points. They hold the relative height and optionally the material of the extension. The sources are stored as type point sources, which broadcast their noise equally in all directions (i.e. omnidirectional). The result after this step is one XML file for each cross-section, since the requirements only allow for one cross-section to be stored in one XML file. These XML files are to be read by the Test_Cnossos software to compute the noise levels at a receiver position. This is described in the next Section. To give an indication of the processing complexity, for a small test area with about 1000 receiver points (see Figure 15) approximately 100.000 cross sections are generated.

#### 3.1.5. Determining noise levels

Test_Cnossos is used to determine the noise level at each receiver point from the XML file containing the noise propagation paths for each receiver point. Noise levels for each receiver point are based on individual paths for that receiver. An energetic summation is carried out to get the total noise level in the 2 km-radius area for one receiver.

In the proof of concept, a standard noise level was assigned to each source (i.e. line segment of the road) and scaled to the length of that specific road segment. The emission height is set at 0.05 m, in line with the CNOSSOS-EU guidelines for traffic. The receiver points are located at a height of 2 m, which is the minimal height for reliable results in CNOSSOS.

The following pipeline is used by Test_Cnossos to generate the noise level at each receiver:

1. Go over each XML file and extract the A-weighted equivalent noise level (LeqA) value
2. Energetically sum all LeqA values for each receiver respectively
3. Create a shapefile containing the 2D receiver points with the calculated noise level as attribute

The result is a shapefile with each receiver point having a noise level value. A noise map can be created by interpolation of these noise levels.

#### 3.1.6. Implementation

Five algorithms have been developed in the Python programming language to implement the above mentioned methodology:

- Algorithm 1: Find noise sources, executed for each receiver
- Algorithm 2: Detect and add reflection points to find a first-order reflection path for each source-receiver pair. Repeat for each source – receiver pair.
- Algorithm 3: Implement straight-walk Algorithm
- Algorithm 4: Extract cross-section
- Algorithm 5: Prepare and write cross-sections to xml files

The source code of these algorithms as well as documentation are available on Github (Dinklo et al. 2020b) and the repository of Delft University of Technology (Dinklo et al. 2020a).

R-Trees have been used to increase the performance of the algorithms. The receiver points cover a large area. However, while dealing with an individual receiver point, only a subset of the overall data is required. The building and road data sets are indexed through the use of R-trees to quickly retrieve the necessary data for a given receiver point.

The time required to run the whole workflow for a test scenario of 100 m by 100 m (Test Scenario 2 in Section 3.2.3) from finding the noise sources to generating the propagation paths and writing these into an XML file, is 48 min (using one core on a regular laptop). The Test_Cnossos software for the same test area takes 7 h to process the 100.000 generated paths. Specifically, the writing of paths to XML as well as writing the computed noise levels to Shape files is time-consuming.

### 3.2. Evaluation method of the prototype: proof of concept

To evaluate if noise simulations can indeed directly be done on a TIN, the work was validated in three different ways:

- An initial visual inspection of the generated cross-sections was carried out, see Section 3.2.1.
- Validation of the XML encoding of the generated cross-sections was run through Test_Cnossos. See Section 3.2.2.
- Both qualitative and quantitative comparisons were conducted between noise maps obtained with the prototype and noise maps obtained with the existing line-based method for two case studies. See Section 3.2.3.

#### 3.2.1. Initial visual inspection of the cross-sections

The created cross-sections were visualized with Rhino (Mcneel, Robert, and Associates n.d.) to interactively validate that the cross-sections indeed adequately represent the DSM representing noise propagation. This was also a good debugging tool especially for reflection paths that require connecting two origin-destination segments as in the first-order reflection paths: receiver point to reflection point and then reflection point to source point. Results are described in Section 4.1.1.

#### 3.2.2. Validation of XML encoding in Test_Cnossos

Test_Cnossos provides a feature that validates the input files before running the software. It checks whether it can access the provided.xml file and whether the file is in accordance with the xml syntax in general. Then, it verifies if all mandatory tags are present and in order, if the tag values are within the range they may fall and are of the right type. At last, it validates the path itself, for example whether a reflection wall allows for a specific reflection, which is required according to the ISO 9613–2 method (van Maercke 2012, 23,24/147). This validation functionality was used to check the generated cross sections encoded in the prescribed XML format. Results are described in Section 4.1.2.

#### 3.2.3. Comparison of noise maps generated with different methods for two test scenarios

The ultimate test of the developed method is to compare the noise levels it produces with results obtained from current line-based simulation software.

The best way to validate the results would have been to compare the calculated noise levels with field measurements of noise levels. However, the validation of calculated noise levels caused by a specific noise source using field measurements is complex. This is due to highly dynamic traffic and weather conditions and the occurrence of other noise sources which are not included in the simulation such as a passing airplane. To address these variances requires field measurements over longer periods, which was outside the scope of this study. Therefore, the results of this research were validated by comparing them to noise levels calculated by a line-based noise simulation.

It was not possible in the scope of this research to implement a line-based CNOSSOS noise simulation method. To be able to compare the results with a line-based method, noise levels were therefore obtained from the existing commercial noise simulation software GeoMilieu. This software implements the *Standaad Reken- en Meetvoorschrift Geluid* (RIVM 2012) that takes height lines as input. The used calculation method is for a large extent similar as the Test_Cnossos method, although differences in the implemented noise simulation methods can cause differences in the outcome. These differences are taken into account during evaluation.

The input data for both simulations are kept as identical as possible to be able to make an appropriate comparison. A simple case was used for testing where all roads have same traffic types and intensities. The height lines used for the GeoMilieu implementation were obtained by converting the triangle boundaries of the TIN into lines. Receiver points were located in a grid of 2 × 2 m. The sample data for roads are taken from TOP10NL, the database underlying the 1:10k map maintained by Kadaster. It is openly available at the national geoportal. The terrain model, LoD1.3 building models and data describing the noise reflective/absorptive characteristics of the terrain are openly available at 3D geo-information (2020).

**Figure 14.**
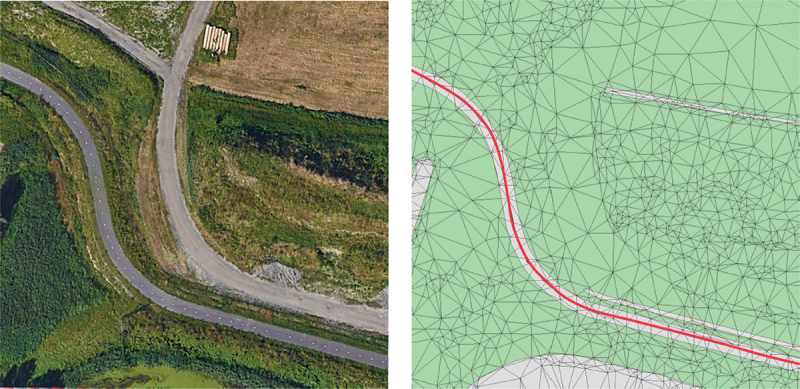
Scenario 1: rural area.

**Figure 15.**
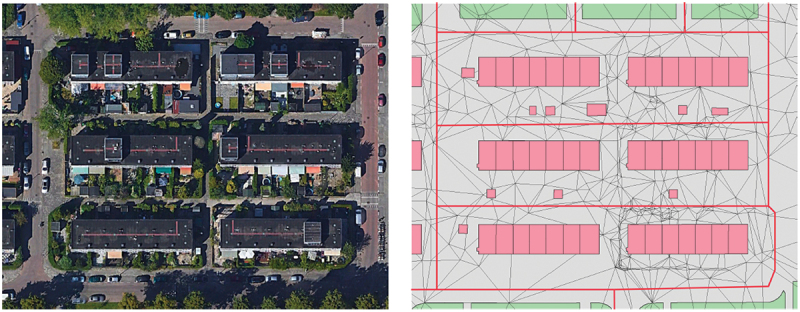
Scenario 2: small suburban block.

Noise maps were produced from both the TIN-based results and the line-based results by (linear) interpolations of the calculated noise levels at each receiver point. The noise maps were created for two test scenarios. Both scenarios are located in the city of Rotterdam. The first scenario (Figure 14) is a large area located outside the city center without buildings. It is a simple scenario: it only contains direct propagation paths, which makes it easy to compare the two methods. The second scenario (Figure 15) is a small suburban building block with road segments. It contains first-order reflections via the buildings. It should be noted that the two scenarios are rather simple and are not equal in size to what is commonly used in a real-world noise study. This is because this research aimed at proofing the concept of TIN-based noise simulation. In future work, the algorithms can be further developed to be optimized and implemented in noise simulation software so that they can be applied to larger areas.

The noise maps were compared both qualitatively and quantitatively. The qualitative evaluation aimed at highlighting the visual differences of the noise maps. The quantitative evaluation is based on calculated differences using map algebra to see if both maps generally have the same noise propagation tendencies and if there were significant differences at specific locations. An absolute power level difference map was made subtracting from each pixel-value in the TIN-based maps the corresponding pixel-value in the line-based method generated maps. This shows how far the results obtained with the developed method are from the line-based method. To complete the quantitative evaluation, the relative noise power level differences were also calculated by extracting for each pixel the line-based method result from the prototype result and dividing this number by the line-based method result. This normalized value ranging from 0 to 100 better shows the locations of significant differences. Due to the earlier mentioned differences between the TIN-based and line-based method and the fact that line-based method cannot be used as ground truth, the quantitative analysis cannot be used in an absolute manner, only to observe trends and major differences.

Results of the qualitative and quantitative evaluation are described, respectively, in Section 4.1.3 and Section 4.1.4.

## 4. Results and discussion

This section presents the results of the evaluations that were performed to validate the proof of concept (Section 4.1). If and how these results prove the concept of TIN-based noise simulation is discussed in Section 4.2.

### 4.1. Results and evaluation

#### 4.1.1. Initial inspection of generated cross sections

The inspection showed that some of the generated reflection cross-sections seem to present spikes at the reflection point. A further investigation into this problem reveals a precision error between two data sets. The semantic constrained TIN provides coordinates with up to three digits, whereas the buildings shape file provides coordinates with up to six digits. This precision discrepancy causes spikes at the reflection point in cross-sections because it does not correctly identify the triangle in which the reflection point exists. After introducing a tolerance, most spikes were resolved and it was decided to leave the remaining spikes for this stage of the project.

#### 4.1.2. Validation of generated.XML files containing propagation paths

In general, the generated files were all valid. Only in the larger tests a few minor errors occurred, and it was decided to ignore those files since it would not have a major impact on the results.

#### 4.1.3. Qualitative comparison between TIN-based and line-based noise maps for two scenarios

To visually assess and compare the results, the values of all generated noise maps are presented by a color ramp ranging from 30 dB(A) to 79 dB(A).

**Figure 16.**
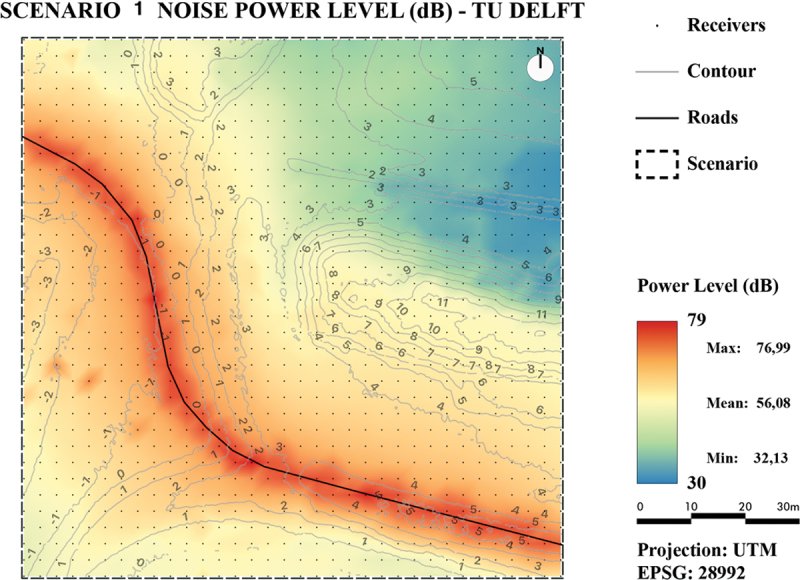
Noise map of Scenario 1 using our developed method.

As can be observed from the maps in Figure 16 for Scenario 1 and Figure 18 for Scenario 2, the noise levels in both areas generated with the TIN-based method behave as expected: They are the highest near the sources and decrease further away from the source. In addition, noise levels surrounding buildings are higher due to reflections on buildings, which results in contour lines that are pulled toward the buildings.

**Figure 17.**
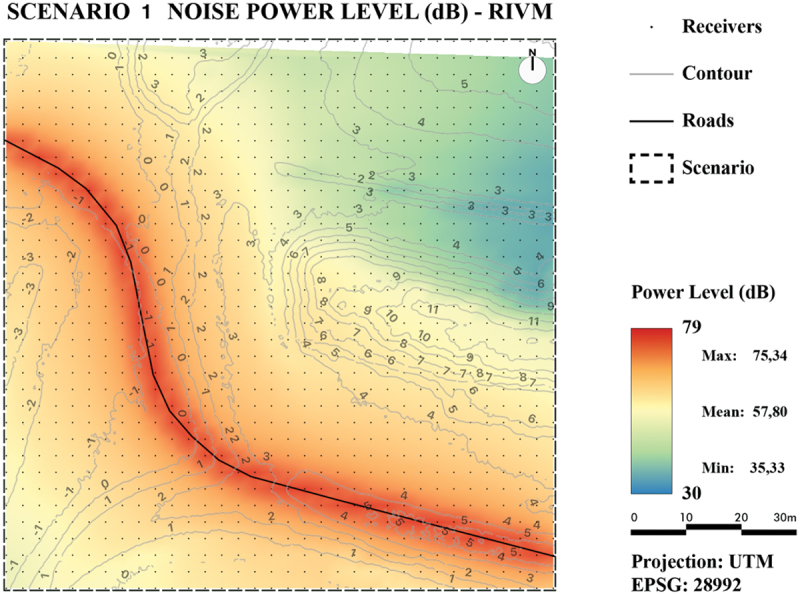
Noise map of Scenario 1 using the line-based method RIVM.

For Scenario 1, the maps in Figure 16 (TIN-based method) and in Figure 17 (line-based method) show that both methods produce similar noise levels. However, the TIN-based method seems to have more local minima and maxima, whereas the line-based method seems to produce more continuous results. This could be explained because the TIN-based method is able to consider more height details.

**Figure 18.**
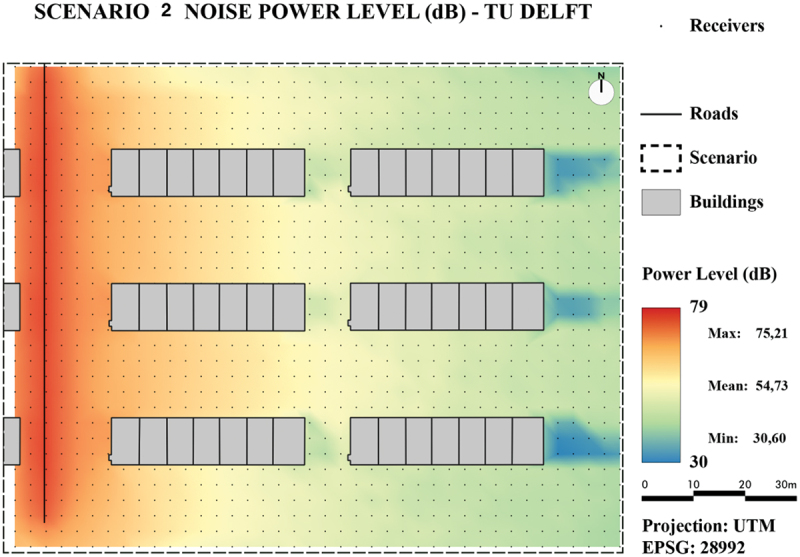
Noise map of Scenario 2 using our developed method.

**Figure 19.**
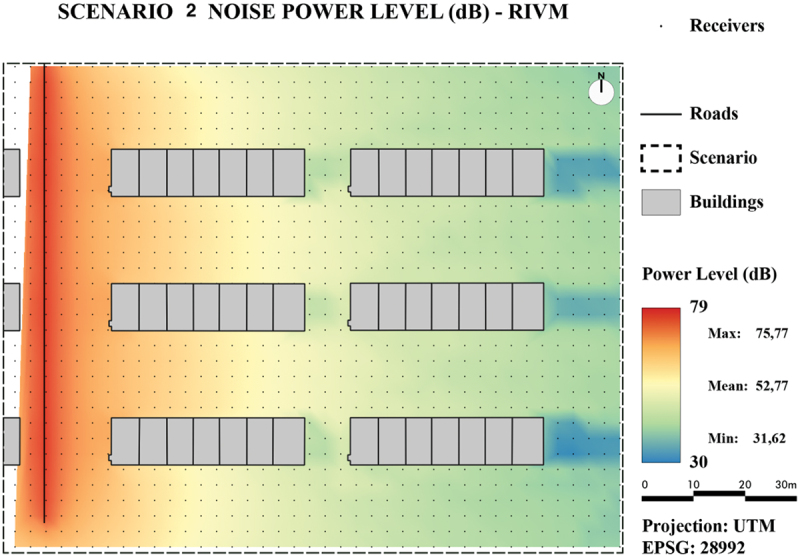
Noise map of Scenario 2 using the line-based method RIVM.

From the noise maps of Scenario 2 containing first-order reflections (Figure 18 (TIN-based method) and Figure 19 (line-based method)), it can be observed that for flat reflecting areas the line-based method shows lower noise levels further away from the source. An explanation could be that a longer distance is traveled in reflections and the details from the TIN adds up and creates higher noise levels at receiver points.

As mentioned before, the noise calculation following SRM2 and CNOSSOS are not exactly the same and these differences should be considered when comparing the two noise maps. With this respect, both scenarios show that CNOSSOS puts more weight to the ground type effect than SRM2.

#### 4.1.4. Quantitative comparison between TIN-based and line-based noise maps for two scenarios

**Figure 20.**
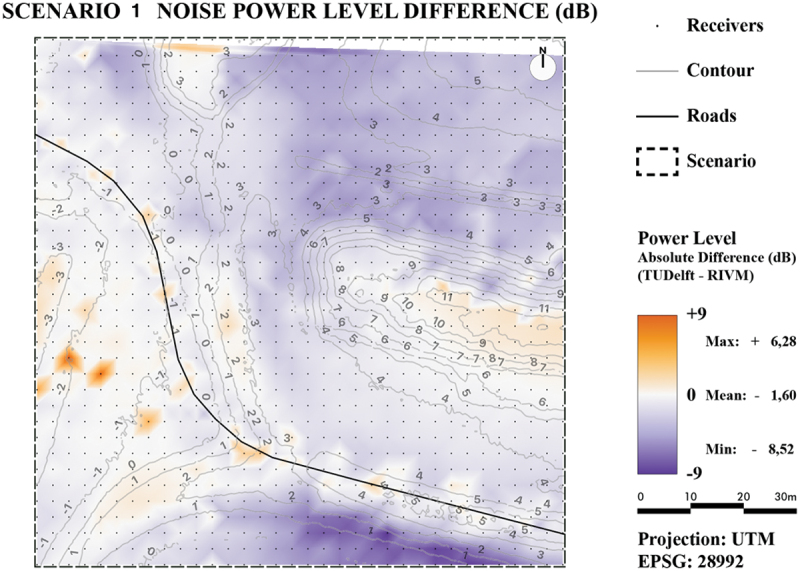
Noise level difference in dB - Scenario 1.

**Figure 21.**
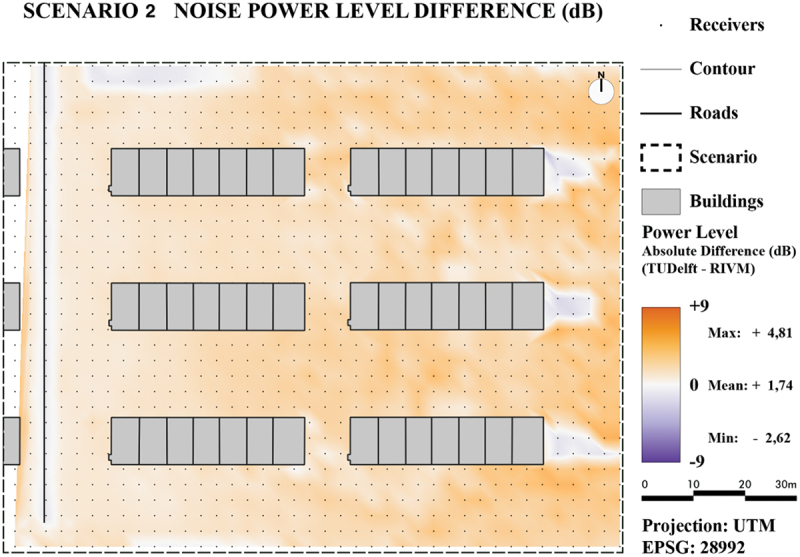
Noise level difference in % - Scenario 1.

**Figure 22.**
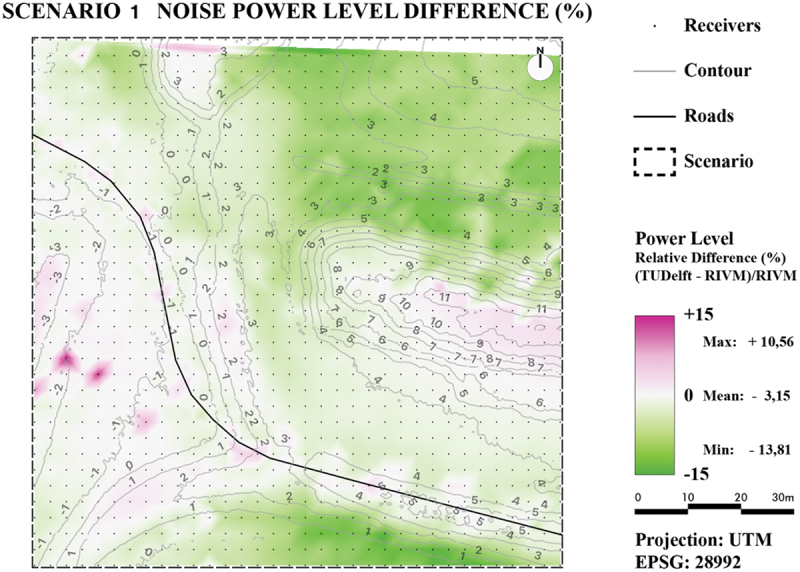
Noise level difference in dB - Scenario 2.

**Figure 23.**
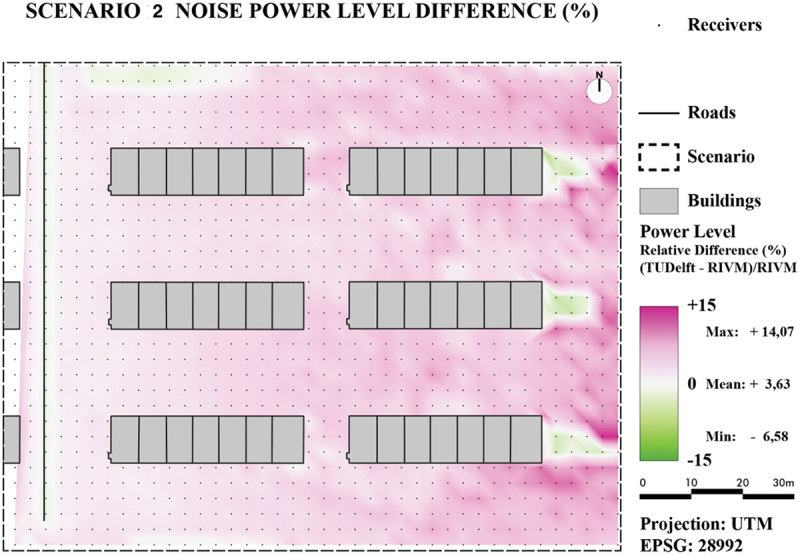
Noise level difference in % - Scenario 2.

For the quantitative comparison, for both scenarios two maps were generated: one with absolute noise value difference (Figure 20 for Scenario 1 and Figure 21 for Scenario 2) and one with relative noise value differences (Figure 22 for Scenario 1 and Figure 23 for Scenario 2).

For both scenarios, it can be concluded that the majority of pixels are within plus or minus 1 dB. The only major difference between the two scenarios is due to the introduction of reflections in Scenario 2 as was already concluded in the previous section. The difference maps also reveal lower noise levels for TIN-based method in occluded areas for Scenario 2, specifically in the lower-right side. This is most likely caused by the earlier mentioned spikes that are located in this area. These spikes will cause the reflection to have a smaller impact on the noise level.

Overall, the difference-maps for Scenario 2 confirm that the TIN-based method is more prone to local height differences. The small differences in Scenario 1 can also mostly be attributed to the extra details in between height lines as provided from the TIN.

Although the quantitative analysis should not (only) be used in an absolute manner due to the differences between the line-based SRM2 method and the TIN-based CNOSSOS method, the comparisons does show similar outputs for both approaches. The tests thus confirm that noise modeling can be directly performed on the TIN and this is the first step for implementation of TIN-based noise simulation in software. Further research needs to be done to see how to optimize the TIN-based algorithms to make them suitable for simulation software.

### 4.2. Discussion of the results

This section discusses the main findings (Section 4.2.1) and discusses the limitation of this work which could be topics for further research (Section 4.2.2).

#### 4.2.1. Main findings

Based on the results, it can be concluded that the TIN-based results are similar to the existing line-based methods for noise simulation and thus that noise simulation can indeed be performed directly on a TIN. Noise simulation that are based on TINs has several advantages above the current line-based methods as described in Section 2.2.3. Firstly, it enables to make use of automatically generated TIN-based input data from widely available point clouds which will significantly improve efficiency and reduce costs in the preparation of input data and at the same time it will improve standardization and consistency between different noise studies. Consistency is also controlled between the different data inputs since all required information (buildings, terrain height and ground type) are integrated in one data structure and processed in one step instead of using three separate layers that are used as input in the line-based methods. This integration enables revealing inconsistencies between the three layers as shown in the tests (e.g. the spikes for the buildings) and it is beneficial for computation. Another advantage of the TIN-based method is that the accuracy of noise simulation can be improved since the TIN-based method enables to represent and incorporate more height details than the method that takes height lines as input data. Finally, a TIN enables to control the quality of the input data and to reduce the height data (i.e. removing triangles from the TIN that do not represent significant height details) with known accuracy losses.

Since the developed method implements the CNOSSOS-EU guidelines, it can directly be used in studies required by the European Directive on noise which offers improvements compared to earlier noise modeling studies as described in Section 2.1. The line-based method was used to see if the TIN-based method would produce comparable noise values and noise propagation patterns as in current noise simulation software. However, the line-based method cannot be used as ground truth. Further research can investigate if the identified differences point at decreased or increased quality of the calculated noise levels, also in the context of the improved accuracy that TIN-based calculations provide compared to line-based simulations, like the representation of height details between height lines.

For the purpose of our study, our focus was on relatively simple, straightforward scenarios. Our study, including the implementation in a prototype and applying it to the two case studies, has both proven our hypothesis that noise modeling can be performed directly on a TIN and has also highlighted limitations and further issues (see next Section). More complicated scenarios need to be tested to further develop our method addressing the identified issues and limitations and to embed it in a full noise assessment workflow.

#### 4.2.2. Topics for further research

There are several other topics that require further research.

First, the developed method is limited to direct paths, first-order reflections, and vertical diffraction. These were all modeled in the horizontal plane and are therefore valid in homogeneous conditions. In reality, there are also horizontal diffraction and higher-order reflections. This effect is quite complex to model and is in most cases not required since it has a negligible impact. For that reason, it was not part of this research. It is interesting to take horizontal diffraction and higher-order reflections into account in future projects that require higher accuracy.

In the scope of this research, only the noise produced by traffic was taken into account. Future research could also include noise from railways, industrial buildings and air planes.

The inclusion of all required information in one data structure (TIN) also enables innovations in noise calculation algorithms, such as the inclusion of buildings with their roof shapes. In the current approaches (also in the existing line-based method), buildings are modeled as LoD1.3 block-representation (Biljecki, Ledoux, and Stoter 2016). It would be interesting for further research to integrate buildings with their roof shapes (the so-called LoD2 models). This way, noise propagation calculation can be done based on a truly 3D path-finding algorithm in which slanted roofs are taken into account.

The noise absorption/reflection in this approach is highly simplified. A ground type is either completely reflecting or absorbing. There are no values in between. On top of that, the current algorithm does not take vegetation into account. These aspects can be addressed in future research.

Finally, the study areas in this research were small. It would be interesting to scale the algorithms for larger areas.

## 5. Conclusion and future work

This paper successfully showed the feasibility to use a TIN describing the height of the terrain as input for noise simulations. Such input data can be generated fully automatically in contrast to height lines that are used as input for current noise simulation software. In addition, it preserves height details between height lines.

The developed prototype to proof the concept of TIN-based noise simulation contained several parts, namely generating the TIN including the required semantics; deriving cross-sections representing propagation paths from the TIN; visually checking these cross-sections in Rhinoceros software; checking the validity of these cross-sections in Test_Cnossos software; and finally, running the Test_Cnossos software with these cross-sections to calculate noise levels at receiver points, creating a noise map from these values and comparing it with a noise map that was created by a line-based noise simulation method.

This comparison showed that a semantic TIN can indeed be used directly for 3D noise modeling and behaves similar as line-based simulation. This makes the extra step to generate height lines unnecessary. In addition to the resulting benefits of automation (standardization, cost-reduction, improving reliability), using a TIN gives more accurate cross-sections since it enables to model height details between height lines and to integrate – and thus control the consistency between – the different data layers required for noise simulation. Therefore, the TIN-based method leads to more accurate noise levels.

The noise simulation that was implemented was performed according to the guidelines of CNOSSOS-EU with Test_Cnossos (an open implementation of CNOSSOS-EU) and therefore the results can be used in the whole European Union.

To bring the benefits of TIN-based noise modeling into practice, the proof of concept is just a first step and needs further development. Apart from further development based on more complex scenarios and tests with ground truth measurements, there are other required improvements before our method can be embedded into a full noise assessment workflow. Firstly, the algorithm to derive the cross-section was written in Python. Converting this into a C++ will significantly improve performance, important for commercial uptake. This will also enable to use the generated paths directly in Test_Cnossos instead of using the intermediate storage of the paths in.XML. Writing these intermediate results appeared to be a computer-intensive task. Additional functionalities can also be added, like horizontal diffraction, higher order reflections and noise produced by other sources than traffic. This will make the TIN-based method a feasible alternative for the current line-based methods and enable input data describing the height of the terrain to be generated fully automatically, leading to more consistent and reliable simulation results as well as cost reduction in noise studies.

Alignment between noise simulation methods, on the one hand, and data science-based methods to automatically generate and prepare the required input data on the other hand, as was done in this paper, will open up new possibilities to improve noise simulation. The proof of concept for TIN-based noise modeling as presented in this paper is a first step.

## Data Availability

The source code of these algorithms as well as documentation and data are available on Github (Dinklo et al. 2020b) and the repository of Delft University of Technology (Dinklo et al. 2020a).

## Appendix

### Pseudo-code of the Straight-walk algorithm and the Writing to XML files algorithm

**Input :**

• origin – A 2D origin point

• destination – A destination point

• current_tr – A triangle that contains the receiver

**Output :**

• next edge that intersects the origin-destination segment

• next triangle to walk to

**while** *not in* destination_triangle **do**

**for** *each* edge *in* current_tr **do**

 edge_index← index of the current edge;

 vertex_right← first vertex of edge;

 vertex_left← second vertex of edge;

   **if** (vertex_right *on the right side of* origin-destination *segment OR on* origin-destination *segment) AND* vertex_left *on the left side of* origin-destination *segment* **then**

   return edge AND edge_index

   **end**

**end**

**end**

**Algorithm 1**: Straight-walk Algorithm adapted from (Zemek 2014)

**Input :**

• vts – A list with succeeding vertices in Cartesian space

• mat – A list with the material of each matching vertex

• ext – A dictionary where key is vts id and value is the extension type and information

• A dictionary with the source noise settings

**Output**: None (writes output xml file)

vts← subtract the source (first point) from each vertex to make the path local (relative to the source) *(the source therefore becomes [0, 0, 0])*;

**if** min(vts.z)<0 **then**

 vts← add min(vts.z) to lift the path such that all vertices have a positive height;

**end**

path← initiate the xml tree with standard CNOSSOS setup, meteo, method and validation settings;

source_noise_level← adapt the default noise levels to the source length;

path← insert all control points (vts) and their extensions;

Write the xml tree to “path_i_j.xml” where:

i← the receiver number;

j← the cross-sections number within the receiver;

**Algorithm 2**: Prepare and write cross-sections to xml files.

## References

[cit0001] 3D geo-information. 2020. “3D Input Data for Noise Studies Version (0.3 February 2020).” Accessed September 15, 2023. https://3d.bk.tudelft.nl/opendata/noise3d/en.html.

[cit0002] Beran, D., K. Jedlička, K. Kumar, S. Popelka, and J. Stoter. 2021. “The Third Dimension in Noise Visualization–A Design of New Methods for Continuous Phenomenon Visualization.” *The Cartographic Journal* 59 (1): 1–17. 10.1080/00087041.2021.188945010.1080/00087041.2021.1889450.

[cit0003] Biljecki, F., H. Ledoux, and J. Stoter. 2016. “An Improved LOD Specification for 3D Building Models.” *Computers, Environment and Urban Systems* 59:25–37. 10.1016/j.compenvurbsys.2016.04.005.

[cit0004] Bocher, E., G. Guillaume, J. Picaut, G. Petit, and N. Fortin. 2019. “NoiseModelling: An Open Source GIS Based Tool to Produce Environmental Noise Maps.” *ISPRS International Journal of Geo-Information* 8 (3): 130. 10.3390/ijgi803013010.3390/ijgi8030130

[cit0005] Cnossos-EU-SWE. 2020. https://github.com/genell/Cnossos-EU-SWE.

[cit0006] Dinklo, C., D. Giannelli, L. van Rijssel, M. Prust, and N. Hobeika. 2020a. “3D Noise Modelling.” Accessed September 15, 2023. http://github.com/Constantijn-Dinklo/3D_Noise_Modelling/tree/master/code.

[cit0007] Dinklo, C., D. Giannelli, L. van Rijssel, M. Prust, and N. Hobeika. 2020b. “3D Noise Simulation.” https://repository.tudelft.nl/islandora/object/uuid:9e83e3c1-0d7b-4026-a34c-2fbb61aaec2c?collection=education.

[cit0008] DIRECTIVE 2002/49/EC. 2002. “DIRECTIVE 2002/49/EC of the EUROPEAN PARLIAMENT and of the COUNCIL of 25 June 2002 Relating to the Assessment and Management of Environmental Noise.” https://eur-lex.europa.eu/legal-content/EN/TXT/PDF/?uri=CELEX:32002L0049from=EN.

[cit0009] European commission. n.d. “Common Noise Assessment Methods in Europe (CNOSSOS-EU).” Accessed September 15, 2023. https://publications.jrc.ec.europa.eu/repository/handle/JRC72550.

[cit0010] Farcas, F., and A. Sivertun. 2012. “Road Traffic Noise: Gis Tools for Noise Mapping and a Case Study for Skåne Region.” *International Archives of the Photogrammetry, Remote Sensing & Spatial Information Sciences* 34.

[cit0011] Kumar, K., A. Labetski, H. Ledoux, and J. Stoter. 2019. “An Improved LOD Framework for the Terrains in 3D City Models.” *ISPRS Annals of the Photogrammetry, Remote Sensing & Spatial Information Sciences* IV-4/W8:75–82. 10.5194/isprs-annals-IV-4-W8-75-201910.5194/isprs-annals-IV-4-W8-75-2019.

[cit0012] Kumar, K., H. Ledoux, T. Commandeur, and J. Stoter. 2017. “Modelling Urban Noise in CityGML Ade: Case of the Netherlands.” *ISPRS Annals of the Photogrammetry, Remote Sensing & Spatial Information Sciences* IV-4/W5:73–81. 10.5194/isprs-annals-IV-4-W5-73-2017.

[cit0013] Kumar, K., H. Ledoux, R. Schmidt, T. Verheij, and J. Stoter. 2020. “A Harmonized Data Model for Noise Simulation in the EU.” *ISPRS International Journal of Geo-Information* 9 (2): 121. 10.3390/ijgi9020121.

[cit0014] Ledoux, H., K. Arroyo Ohori, R. Peters, and M. Pronk. 2020. “Computational Modelling of Terrains.” https://github.com/tudelft3d/terrainbook.

[cit0015] Licitra, G. 2012. *Noise Mapping in the EU: Models and Procedures*. Boca Raton, FL: CRC Press, Taylor & Francis Group.

[cit0016] Mcneel, Robert and Associates. n.d. “Rhinoceros Rhino 6 CAD Software.” Accessed September 15, 2023. https://www.rhino3d.com.

[cit0017] Pervez, A., K. Ahmad, S. S. Afsar, and N. Akhtar. 2020. “Noise Monitoring, Mapping, and Modelling Studies – a Review.” *Journal of Ecological Engineering* 21 (4): 82–93. 10.12911/22998993/119804.

[cit0018] Picaut, J., N. Fortin, E. Bocher, G. Petit, P. Aumond, and G. Guillaume. 2018. “An Open-Science Crowdsourcing Approach for Producing Community Noise Maps Using Smartphones.” *Building and Environment* 10. 10.1016/j.buildenv.2018.10.049.

[cit0019] RIVM. 2012. “Standaard Reken- en meetvoorschrift geluid 2012.” Accessed September 15, 2023. https://wetten.overheid.nl/BWBR0031722/2022-10-01.

[cit0020] RIVM. 2014. “Standaard Reken- en meetvoorschrift geluid 2012.” Accessed September 15, 2023. https://www.rivm.nl/bibliotheek/rapporten/2014-0127.pdf.

[cit0021] Stoter, J., H. Kluijver, and V. Kurakula. 2008. “3D Noise Mapping in Urban Areas.” 10.1080/1365881070173903910.1080/13658810701739039.

[cit0022] Stoter, J., R. Peters, T. Commandeur, B. Dukai, K. Kumar, and H. Ledoux. 2020. “Automated Reconstruction of 3D Input Data for Noise Simulation.” http://www.sciencedirect.com/science/article/pii/S0198971519302662.

[cit0023] United Nations. 2018. “68% of the World Population Projected to Live in Urban Areas by 2050, Says UN.” May. https://www.un.org/development/desa/en/news/population/2018-revision-of-world-urbanization-prospects.html.

[cit0024] van Maercke, D. 2012. “Task 2: Propagation software modules; User’s and programmer’s guide.” Company: Centre Scientifique et Technique du Bâtiment (CSTB). unpublished comes with TestCnossos software; requests mailed to: dirk.van-maercke@cstb.fr.

[cit0025] Zemek, M. 2014. “Regular Triangulation in 3D and Its Applications.” https://www.researchgate.net/publication/242533218_Regular_Triangulation_in_3D_and_Its_Applications.

[cit0026] Zhao, W.-J., E.-X. Liu, H. Joo Poh, B. Wang, S.-P. Gao, C. Eng Png, L. Kelvin Wenhui, and S. Hao Chong. 2017. “3D Traffic Noise Mapping Using Unstructured Surface Meshrepresentation of Buildings and Roads.” techreport 127. Singapore: Institute of High Performance Computing, A*STAR. 10.1016/j.apacoust.2017.06.025.

